# Risk factors and spatio-temporal patterns of livestock anthrax in Khuvsgul Province, Mongolia

**DOI:** 10.1371/journal.pone.0260299

**Published:** 2021-11-19

**Authors:** Tuvshinzaya Zorigt, Satoshi Ito, Norikazu Isoda, Yoshikazu Furuta, Misheck Shawa, Natsagdorj Norov, Baasansuren Lkham, Jargalsaikhan Enkhtuya, Hideaki Higashi

**Affiliations:** 1 Division of Infection and Immunity, International Institute for Zoonosis Control, Hokkaido University, Sapporo, Japan; 2 Unit of Risk Analysis and Management, International Institute for Zoonosis Control, Hokkaido University, Sapporo, Japan; 3 Laboratory of Microbiology, School of Veterinary Medicine, Hokkaido University, Sapporo, Japan; 4 Division of Quality Management and Coordination, Mongolian University of Life Sciences, Ulaanbaatar, Mongolia; 5 Laboratory of Infectious Disease and Immunology, Institute of Veterinary Medicine, Mongolian University of Life Sciences, Ulaanbaatar, Mongolia; 6 Laboratory of Food Safety and Hygiene, Institute of Veterinary Medicine, Mongolian University of Life Sciences, Ulaanbaatar, Mongolia; USGS WCWRU: US Geological Survey Wisconsin Cooperative Wildlife Research Unit, UNITED STATES

## Abstract

Anthrax is a worldwide zoonotic disease. Anthrax has long been a public health and socio-economic issue in Mongolia. Presently, there is no spatial information on carcass burial sites as a potential hazard of future anthrax outbreaks and possible risk factors associated with anthrax occurrences in Mongolia. Here, we analyze retrospective data (1986–2015) on the disposal sites of livestock carcasses to describe historical spatio-temporal patterns of livestock anthrax in Khuvsgul Province, which showed the highest anthrax incidence rate in Mongolia. From the results of spatial mean and standard deviational ellipse analyses, we found that the anthrax spatial distribution in livestock did not change over the study period, indicating a localized source of exposure. The multi-distance spatial cluster analysis showed that carcass sites distributed in the study area are clustered. Using kernel density estimation analysis on carcass sites, we identified two anthrax hotspots in low-lying areas around the south and north regions. Notably, this study disclosed a new hotspot in the northern part that emerged in the last decade of the 30-year study period. The highest proportion of cases was recorded in cattle, whose prevalence per area was highest in six districts (i.e., Murun, Chandmani-Undur, Khatgal, Ikh-Uul, Tosontsengel, and Tsagaan-Uul), suggesting that vaccination should prioritize cattle in these districts. Furthermore, size of outbreaks was influenced by the annual summer mean air temperature of Khuvsgul Province, probably by affecting the permafrost freeze-thawing activity.

## Introduction

Anthrax is a zoonotic disease caused by the Gram-positive bacterium *Bacillus anthracis*, existing as a spore outside its host animal [1]. The spores are highly resistant to extreme temperatures, radiation, and chemical substances and can persist in soil for several decades [2]. Anthrax has a wide range of mammalian hosts, including humans, livestock, and wildlife. Domesticated herbivores such as cattle, sheep, and goats, are the most susceptible to anthrax; grazing with ingesting or inhaling high doses of spores usually culminates in fatal disease [3]. Anthrax is not contagious as direct transmissions between herbivores are thought to be rare [4,5]. Although herbivores are the primary hosts for anthrax, humans also get infected with anthrax through contact with infected animals or contaminated animal products, such as meat, hide, and wool [6].

Several environmental factors influence the persistence of *B*. *anthracis* spores and the onset of anthrax outbreaks. Long-range dispersal of spores is influenced by weather conditions, such as floods and strong winds [7,8], and by biological vectors, such as birds, scavengers, and biting flies [9–11]. Soil conditions, such as high moisture, rich calcium, high organic content, and pH neutral to alkaline, correlate with the onset of outbreaks [12,13]. Spores can be concentrated in low-lying areas through runaway by rain and water streams, affecting the spatial distribution of outbreaks [14]. Recent studies highlight the role of permafrost in preserving *B*. *anthracis* spores for a long time in the frozen ground below 0°C, and the effect of increasing temperature on permafrost thawing due to the spore spillover to the soil surface [15].

Anthrax is globally distributed, and sporadic cases have been reported on every populated continent [16]. In industrialized countries where focused, comprehensive, and sustained livestock vaccination programs have been successfully implemented, the disease has dramatically declined [17]. However, the burden of anthrax remains high in low- and middle-income countries in Africa and Asia, where animal vaccination is erratic [18].

In Mongolia, anthrax has posed severe challenges to public health and veterinary services for a long time. Approximately 25% of the national population of 3,000,000 residents live a nomadic pastoral lifestyle, raising livestock under a free-ranging system. Livestock products are an essential income source for these people [19], thus, making anthrax control a great priority. Mongolia was once a socialist country with close connections to the Soviet Union from around 1920 to the late 1980s. The introduction of routine animal vaccination from 1948 onward resulted in a drastic decrease in anthrax incidence [20]. However, following the collapse of the Soviet Union, political revolution and economic transition from socialism to a free market began in the early 1990s in Mongolia. Due to the transformation recession, healthcare delivery and mass vaccination in the veterinary sector were ceased [21]. Among 19 of 21 provinces in Mongolia, with records of anthrax incidences, frequent outbreaks are restricted to the provinces at the northern and northeastern regions of the country [20]. Among them, Khuvsgul has the highest livestock anthrax rate, accounting for 40.35% of all cases [22].

The spores of *B*. *anthracis* can persist in soil for several decades, and may even be preserved longer in frozen grounds [23]. The potential hazard of the historic carcass sites was confirmed by surveys of viable spore detection in previous carcass sites [9]. The current disposal practice of animals dying from anthrax in Mongolia mainly involves burying, and those carcass burial grounds are generally identical to outbreak locations [24]. Although disease control measures include restriction of access to carcass burial sites, which are most likely to be contaminated with spores, presently, there is a lack of information on the spatial pattern of carcass sites, thus limiting the implementation of interventions. Here, we analyze retrospective data (1986–2015) on the burial sites of livestock carcasses to describe historical spatial patterns and temporal trends in livestock anthrax across the Khuvsgul Province to answer the following questions; What is the spatial pattern of carcass sites? Where are the areas of high carcass site concentration in previous and recent times? Which districts have more burden of anthrax? Also, we were interested in exploring the relationship between the number of anthrax cases and animal population, annual mean precipitation, and annual mean summer air temperature, considering the anthrax seasonality in Mongolia [20]. This study is the first spatio-temporal study on anthrax in Mongolia and could serve as a baseline for future anthrax studies and public health interventions.

## Materials and methods

### Data source

Among 21 administrative provinces in Mongolia, Khuvsgul Province is the northmost province, bordering Siberia, Russia, with 24 districts. Anthrax is notifiable under the law on Livestock Health and Gene Protection of Mongolia [24]. Thus, reporting of all suspected cases of anthrax is mandatory. An animal or animal carcass with symptoms suggestive of anthrax (e.g., high fever, breathing difficulty, sudden death, a carcass without rigor mortis, etc.) was considered as a suspicious case, while a positive standard bacteriology and/or molecular laboratory tests was defined as a confirmed case [25]. Here, we collected basic yearly information on livestock in Khuvsgul Province, including livestock and human population, the number of livestock anthrax cases, and GIS data of carcass burial sites from 1986 to 2015. The Department of Veterinary Services of Khuvsgul Province, Mongolia, provided the geographic information system (GIS) data for anthrax carcass disposal sites recorded between 1986 and 2015. All these sites represent confirmed cases previously diagnosed by clinical examination and standard bacteriology method at the local veterinarian and the State Central Veterinary Laboratory (SCVL), Ulaanbaatar, Mongolia. The standard bacteriology methods included Giemsa and polychrome methylene blue (PMB) staining for microscopic detection of *B*. *anthracis* and its poly-D-glutamic acid capsule in blood smears. The methods also involved culture and isolation of the bacterium, as previously described in the OIE manual [25]. In addition, samples collected after 2000 were further confirmed by polymerase chain reaction (PCR) for the detection of toxin (*pagA*) and capsule (*capB*) coding genes of *B*. *anthracis* at the SCVL. This improvement in disease diagnosis would not significantly impact the anthrax surveillance since the disagreement between the standard bacteriology and PCR tests for anthrax confirmation is negligible [26].

The livestock and human population data in the Khuvsgul Province was obtained from the Statistics Office of Khuvsgul Province, Mongolia [27]. Data on annual precipitation and air temperature of Khuvsgul Province were provided by the Information and Research Institute of Meteorology, Hydrology, and Environment, Mongolia. Administrative areas, elevation, and inland water maps of Mongolia were downloaded from database of DIVA-GIS [28]. The project was approved by the review board of the Institute of Veterinary Medicine, Mongolia, Reference number: 20082001.

### The cattle anthrax prevalence per area in Khuvsgul Province by districts (1986–2015)

The annual average prevalence of anthrax per 1,000,000 cattle population per 1000 km^2^ was calculated at the district level. *B*. *anthracis* is a non-invasive pathogen and regarded as non-contagious; therefore, direct animal-to-animal transmission is not expected to occur except in osteophagia or carnivore activities [4,29]. Indeed, soil is the natural reservoir of the *B*. *anthracis* spores and becomes the primary source of animal infection [30]. Thus, considering the mode of anthrax transmission, we estimated the cattle anthrax prevalence, taking into account the area (km^2^) of each district as follows:

$$prevalence\;per\;area=\frac{annual\;average\;anthrax\;cases}{\left(average\;animal\;population\times area\right)}$$

A cartographic map was used to visualize the distribution of cattle anthrax prevalence using ArcGIS v.10.6.1 (ESRI Inc., Redlands, CA, USA).

### The spatial mean and standard deviational ellipse analyses

The analyses were conducted on ArcGIS v.10.6.1 software (ESRI Inc., Redlands, CA, USA) [31,32]. The standard deviational ellipse (SDE) analysis was performed to summarize the spatial attributes of geographic features with coordinates [33]. The unweighted spatial mean and SDE analyses were used to determine the directional trend and spatial characteristics of carcass sites in the study area. To reveal the temporal changes of carcass sites resulting from anthrax incidences, we divided the entire study period into three time parts: 1986–1995, 1996–2005, and 2006–2015.

### Multi-distance spatial cluster analysis

According to the guide on the manufacturer’s website, we used a multi-distance spatial cluster analysis tool in ArcGIS v.10.6.1 software (ESRI Inc., Redlands, CA, USA) to determine the maximum distance relationship between animal carcass sites [34]. The tool uses Ripley’s K function as shown in the equation

$$L(d)=\sqrt{[A\sum_{i=1}^{N}\sum_{j=1,i\neq j}^{N}k(i,j)]/\left[\pi N(N-1)\right]}$$

where d is the distance, N is the total number of events, A is the area, and the weight k(i,j) is the influence of the elements within the distance. When the distance between i and j is less than or equal to d, k(i,j) is 1, and k(i,j) is 0 when the distance between i and j is greater than d.

To analyze the spatial pattern of carcass sites, observed K-values, determined using actual GIS coordinates, were compared with the expected K-values, calculated through the random spatial distribution of carcass sites. We used the default 10 times as the number of distance changes with 999 simulations, equal to confidence levels of 99.9%. We utilized the minimum enclosing rectangle as the study area method. Positive value from the difference between the observed K and expected K (Diff K) indicates clustering. When the observed K-value for a specified expected K-value is larger than the upper confidence envelope (HiConEnv) value, the spatial value is statistically significant. In the following kernel density estimation analysis, to avoid underestimating the hotspot areas, the maximum expected K with a statistically significant value was used as the maximum distance for the relationship between carcass sites in the Khuvsgul Province (S1 Table).

### Kernel density estimation analysis

The kernel density estimation (KDE) analysis was used to identify hotspots of animal carcass disposal locations. The analysis was performed using ArcGIS v.10.6.1 software (ESRI Inc., Redlands, CA, USA) by employing the quadratic kernel function described by Silverman *et al*. [35] to estimate carcass densities. We applied 88, 58, 83, and 88 km, calculated from Ripley’s K function corresponding to the periods (i.e., 1986–2015, 1986–1995, 1996–2005, 2006–2015) as a search radius. The KDE output is classified into five categories, according to the equal interval method.

### Statistical analyses

Univariate and multivariate logistic regression models were used to determine potential risk factors associated with a large number of anthrax cases among livestock in the Khuvsgul Province. In the univariate analysis, anthrax outbreaks were categorized into a large or small number of cases. Annual cases that were above the mean of total cases (≥ 51) during the whole study period (1986–2015) were considered a large number of cases and were separately analyzed with different variables, including total livestock population, cattle population, human population, annual mean air temperature in summer months (June to August), and annual mean precipitation. In Mongolia, anthrax is a seasonal disease that mostly occurs in summer when animals graze on pastures [20]. In addition, most permafrost in the Khuvsgul region is at temperatures close to 0°C, and thaws only in summer [36], which possibly leads to spore spillover from buried carcasses to the soil surface. Thus, we investigated the effect of temperature variability in summer. The variables that met the criteria of *p* < 0.2 in the univariate analysis were further evaluated in the multivariate logistic regression model, with a statistical significance level at *p* < 0.05. Afterward, correlation tests were conducted between anthrax cases and cattle population or temperature to determine the direct relationships.

Simple linear regression analyses were used to determine livestock anthrax trends corresponding to before and after the Mongolian economic transition (1986–2000 and 2001–2015, respectively). A linear regression model was also applied to identify the annual summer (June to August) temperature changes in the Khuvsgul Province throughout the 30-year study period from 1986–2015. All statistical analyses were conducted using R v.3.5.0.

## Results

### Old and recent trends of anthrax between 1986 and 2015

**Fig 1:**
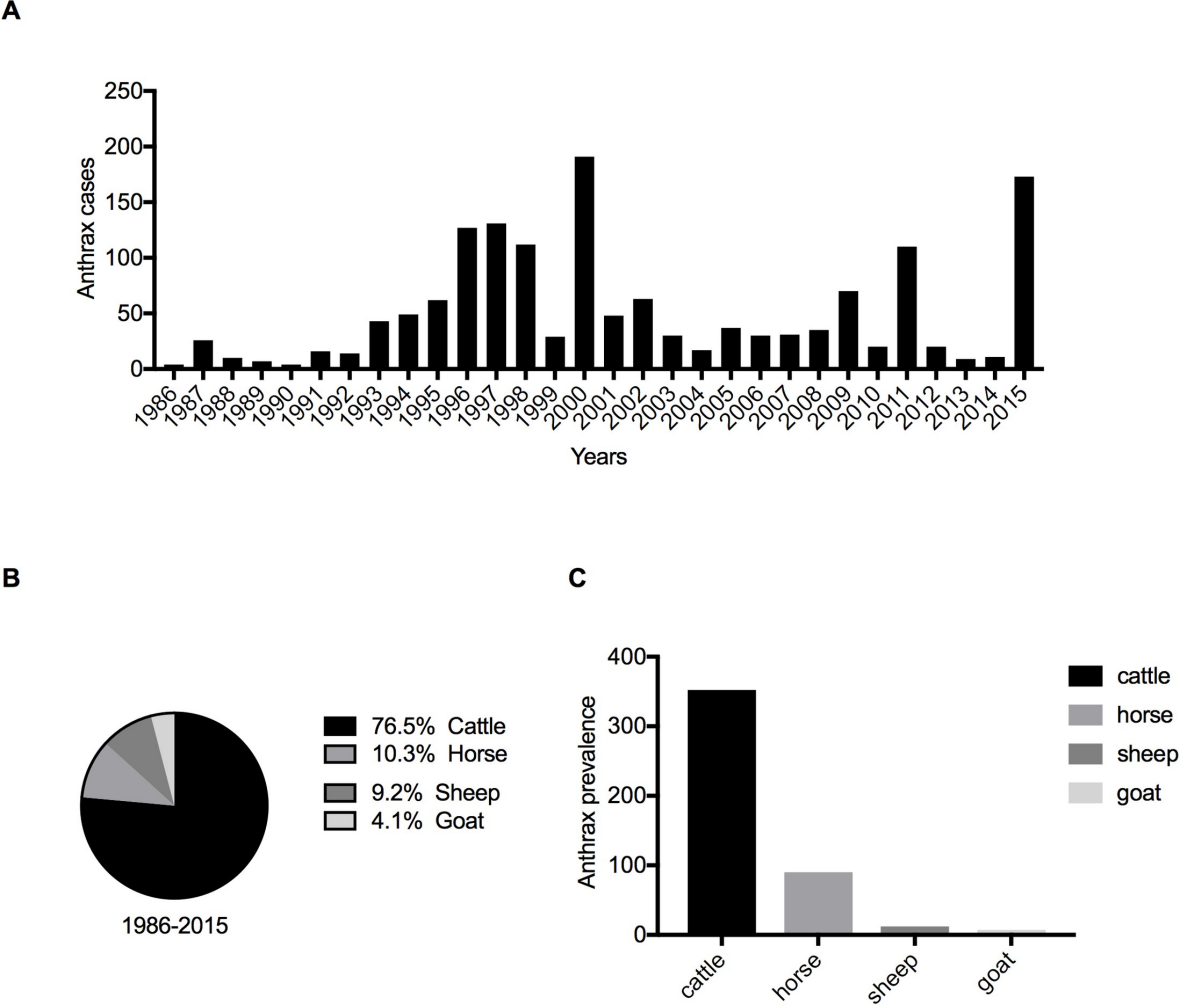
The distribution of anthrax in Khuvsgul Province between 1986 and 2015. (A) Annual dynamics of registered livestock anthrax cases. (B) Reported anthrax cases in livestock species. (C) Prevalence of anthrax in livestock by species for 30 years.

A total of 1529 livestock cases were reported over the study period (1986–2015), with the majority of cases reported in cattle (76.5%; n = 1169), followed by horse (10.3%; n = 157), sheep (9.2%; n = 141), and goat (4.1%; n = 62) (Fig 1A and 1B). Regarding the disease prevalence in livestock by animal species for 30 years, cattle were the highest, followed by horse, sheep, and goat, with proportions of 352, 90, 12, and 4 per 100,000 population, respectively (Fig 1C). There were no anthrax reports in sheep and horses before 2000, and few outbreaks were reported in goats. After 2000, the host range expanded, and reports of anthrax incidents in goats, sheep, and other animal species had increased. Anthrax outbreaks in horses were sporadic and occurred only three times over the entire 30-year period. However, a large outbreak occurred in 2015, affecting more than 150 horses (S1 Fig).

Further, we conducted simple linear regression analyses to determine livestock anthrax trends corresponding to before and after the Mongolian economic transition (1986–2000 and 2001–2015, respectively). A dramatic increase was observed in the annual number of anthrax cases between 1986 and 2000, with an average rate of 10.1 ± 2.3 (*p* < 0.001) in a year. Several large outbreaks occurred after 2000, and the average annual case number was 2.5 ± 2.6 without significant increase or decrease in cases (*p* = 0.35) (Table 1). Although neither a significant increasing nor decreasing trend was observed in the total livestock anthrax cases, cattle anthrax cases were significantly reduced (S2 Table).

**Table 1:** Annual livestock anthrax incidences in Khuvsgul Province during (1986–2000) and after (2001–2015) the economic transition of Mongolia.

				Linear regression		
Period	Anthrax cases in livestock	Ann^1^ min	Ann^2^ max	Slope	*p*	R^2^
1986–2000	825	4	191	10.10 ± 2.261	0.0006	0.6058
2001–2015	704	9	173	2.529 ± 2.622	0.3524	0.06678

^1^Minimum annual number of cases

^2^Maximum annual number of cases.

**Fig 2:**
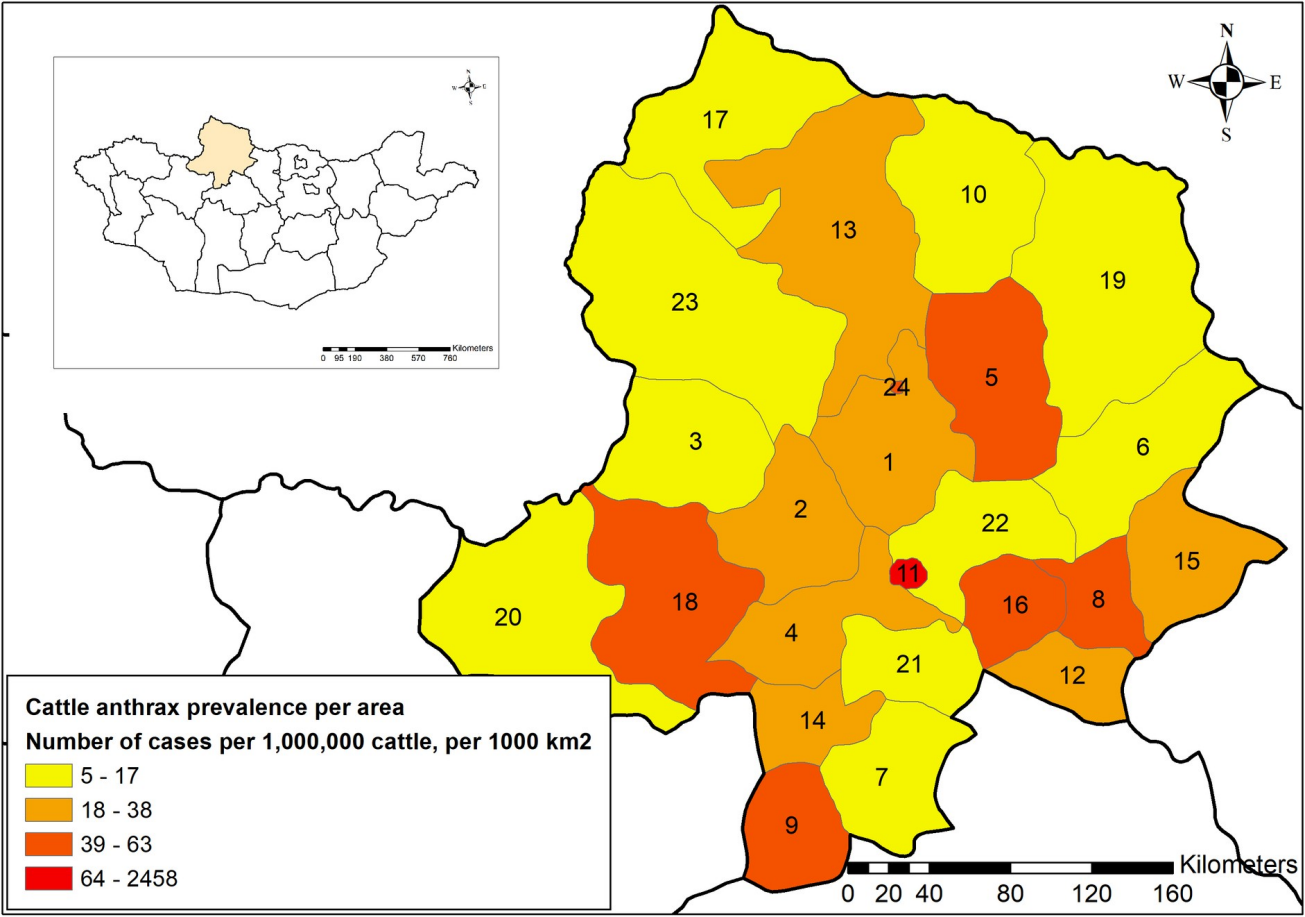
Annual cattle anthrax prevalence per 1,000,000 population per 1000 km^2^ in Khuvsgul Province by districts. Numbers correspond to the ID number in Table 2. The Murun (district ID 11), the central administrative district of Khuvsgul Province, had the highest cattle anthrax prevalence per area. The maps are reprinted from [28] under a CC BY license, with permission from DIVA-GIS and Dr. Robert Hijmans; see S1 File.

A total of 1169 cattle anthrax cases were reported in Khuvsgul Province between 1986 and 2015. Annual cattle anthrax prevalence per 1,000,000 population per 1000 km^2^ was high in the districts Murun (ID 11), Chandmani-Undur (ID 5), Khatgal (ID 24), followed by Ikh-Uul (ID 8), Tosontsengel (ID 16), and Tsagaan-Uul (ID 18) (Table 2 and Fig 2). The average human and livestock population density for 30 years was highest in the Murun district (S2 Fig).

**Table 2:** Annual cattle anthrax prevalence per 1,000,000 population per 1000 km^2^ in Khuvsgul Province by districts.

ID	District name	Number of cattle cases	Average cases (min^a^–max^b^)	Average cattle population (min^c^–max^d^)	Area of the districts (km^2^)	Ann^1^ prevalence
1	Alag-Erdene	59	1.97 (3–29)	12106 (8315–20094)	4503	36
2	Arbulag	45	1.5 (1–28)	16115 (7579–27823)	3529.2	26
3	Bayanzurkh	36	1.2 (2–20)	16275 (11593–23319)	4299.1	17
4	Burentogtokh	66	2.2 (1–26)	15174 (5755–24978)	3768.6	38
5	Chandmani-Undur	120	4 (1–42)	14054 (11159–16975)	4487.5	63
6	Erdenebulgan	34	1.13 (1–18)	13845 (10447–16497)	4694.4	17
7	Galt	32	1.07 (1–12)	17300 (9654–27537)	3596.8	17
8	Ikh-Uul	51	1.7 (1–20)	13709 (9873–18942)	2023.8	61
9	Jargalant	44	1.47 (1–15)	12624 (6100–20375)	2549.2	46
10	Khankh	16	0.53 (1–13)	8589 (903–14919)	5498.7	11
11	Murun	85	2.83 (1–24)	11200 (6207–20150)	102.9	2458
12	Rashaant	19	0.63 (1–3)	12230 (8874–17462)	1982.5	26
13	Renchinlkhumbe	121	4.03 (1–18)	22632 (16469–29020)	8448.3	21
14	Shine-Ider	25	0.83 (1–7)	13897 (7140–24723)	2053.6	29
15	Tarialan	50	1.67 (1–25)	17058 (12000–22176)	3430.7	28
16	Tosontsengel	43	1.43 (1–9)	13592 (8823–21358)	2042.2	52
17	Tsagaan-Nuur	4	0.13 (2)	2602 (835–3991)	5408.3	9
18	Tsagaan-Uul	170	5.67 (2–113)	18345 (4913–37194)	5866.3	47
19	Tsagaan-Uur	21	0.7 (2–12)	12855 (10419–15131)	8735.3	6
20	Tsetserleg	59	1.97 (1–24)	16990 (5967–33153)	7451.6	16
21	Tumurbulag	16	0.53 (1–8)	12603 (6557–19964)	2521.7	17
22	Tunel	16	0.53 (1–4)	12646 (8828–17166)	3577.3	12
23	Ulaan-Uul	27	0.9 (1–18)	18739 (13615–23438)	10057.5	5
24	Katgal	10	0.33 (4–6)	6990 (3915–9657)	911.4	52

^1^Annual anthrax prevalence per 1,000,000 cattle per 1000 km2.

^a^ Minimum annual number of cattle anthrax cases

^b^ Maximum annual number of cattle anthrax cases.

^c^ The smallest annual cattle population number

^d^ The largest annual cattle population number.

### The spatio-temporal anthrax pattern and high-risk areas

**Fig 3:**
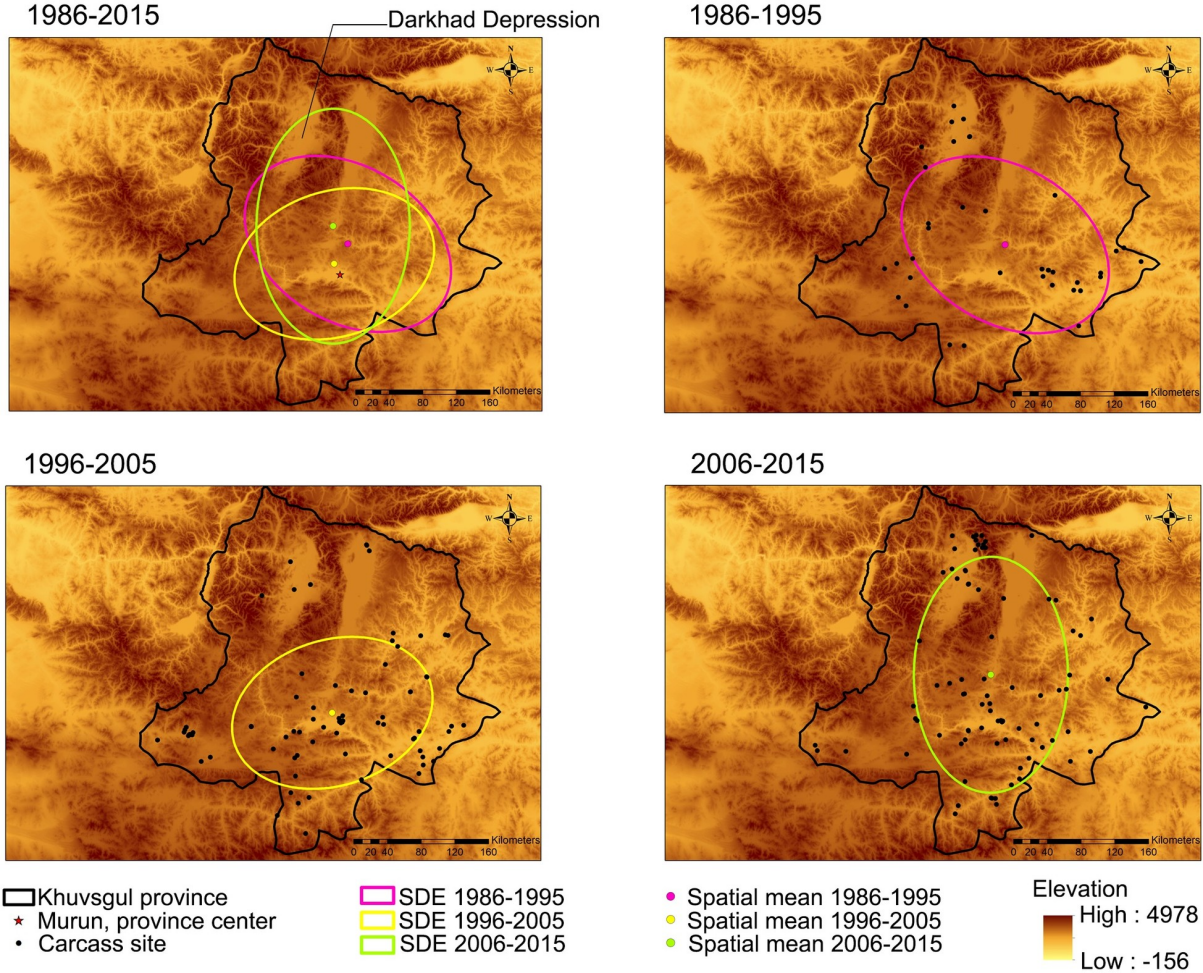
The directional distribution of animal carcass sites registered between 1986 and 2015. Unweighted spatial means and standard deviational ellipse (SDE) were determined in three periods (1986–1995, 1996–2005, and 2006–2015). The unweighted spatial mean centers indicate the average value of carcass locations in the given time phases. Ellipses with mean centers were combined to denote the directional distribution of carcass sites. The base map is the elevation in meters, with lighter areas being lower in elevation. The maps are reprinted from [28] under a CC BY license, with permission from DIVA-GIS and Dr. Robert Hijmans; see S1 File.

The three identified SDEs with their spatial means of carcass locations are shown in Fig 3. There were no significant directional changes in anthrax occurrence between the three periods and the spatial means of carcass sites adjoined in the southern part of the study area. By overlaying the ellipses and spatial means on an elevation map of Khuvsgul Province, it was observed that carcass sites were distributed along the rivers and seasonally dry riverbeds that confluent into wide rivers in the southern region.

To determine the distribution pattern of carcass sites and the maximum distance between carcass sites in the three periods, multi-distance spatial cluster analysis was conducted. The result indicated that the maximum distances of the significant spatial association between carcass sites corresponding to the periods (i.e., 1986–2015, 1986–1995, 1996–2005, and 2006–2015) were 88, 58, 83, and 88 km, respectively (S1 Table). This result showed that carcass sites distributed in the study area are clustered rather than dispersed in the specified range of distances, and the observed relationships were statistically significant.

**Fig 4:**
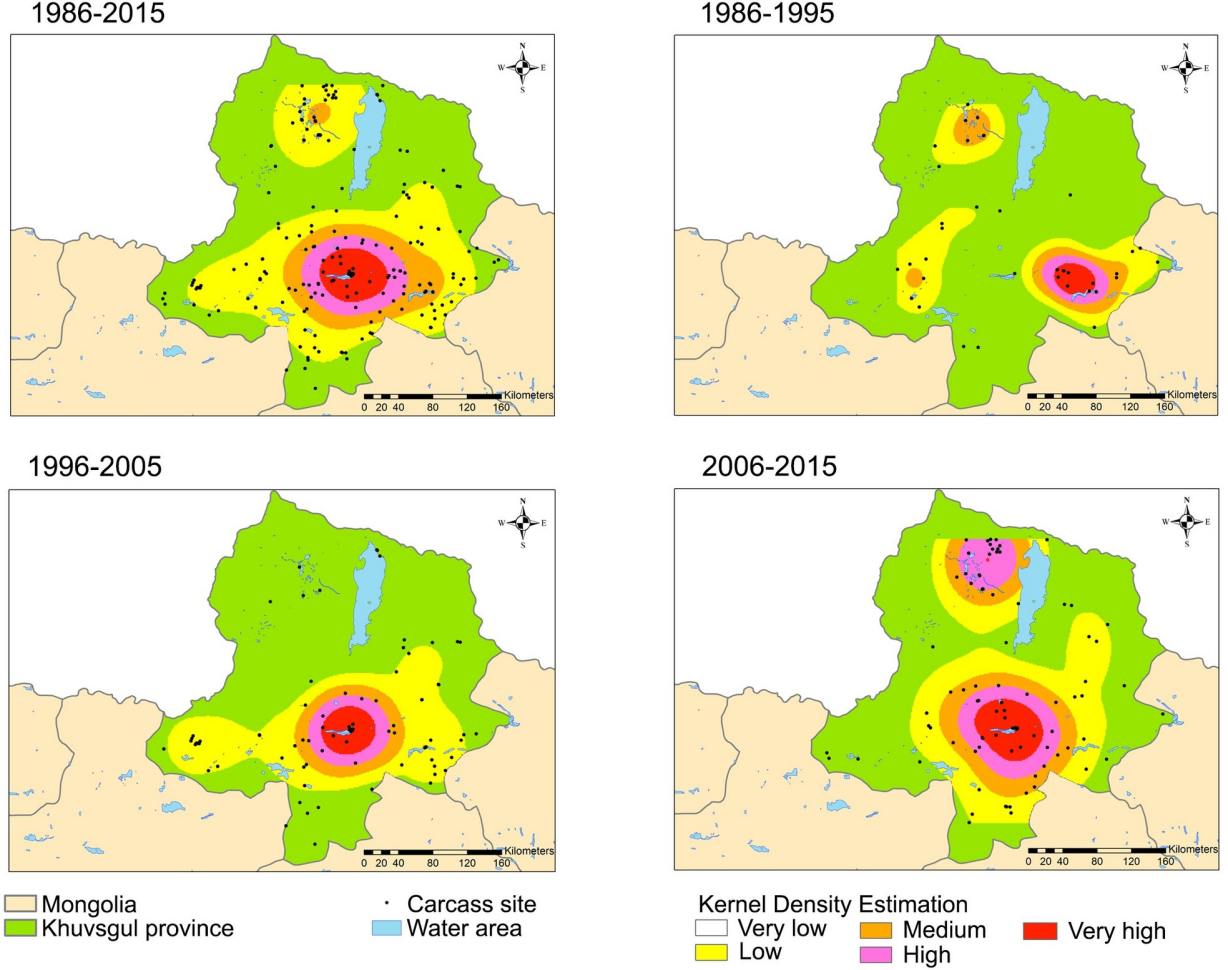
Hotspot analysis of carcass sites using kernel density estimation. The heat maps show the estimated density of anthrax carcass sites per square kilometer from very low (transparent) to very high (red): very low <20%, low 40%, medium 60%, high 80%, and very high >80% of the estimated highest values in each period. The maps are reprinted from [28] under a CC BY license, with permission from DIVA-GIS and Dr. Robert Hijmans; see S1 File.

The identified maximum distances were then used in the KDE analysis to estimate the density of carcasses to identify anthrax hotspots. We found consecutive anthrax hotspots in the southern region of the Khuvsgul Province across the entire study period and an emerging new hotspot in the northern part of the area between 2006 and 2015 (Fig 4).

### Positive association between cattle population, temperature, and anthrax case numbers

Two factors, cattle population number and annual mean temperature of summer months, have met the criteria in the initial univariate analyses. In multivariate logistic regression analysis, the odds of having a large number of anthrax cases (≥ 51) were multiplied by 7.63 for every 100,000 increase in cattle population size and by 2.86 for every 1°C increase in mean temperature of summer (Table 3).

**Table 3:** Factors potentially associated with a large anthrax outbreak occurrence by univariate and multivariate logistic regression: Cases (large > 51 ≥ small) *vs*. factors.

Factors Univariate	OR	95% CI for OR	*p* < 0.2
Total livestock population	1	1.00–1.00	0.5062
Cattle population	5.99	1.53–36.46	0.0224
Human population	1	1.00–1.00	0.273
Annual mean summer temperature (Jun-Aug)	2.07	1.02–5.11	0.0661
Annual mean precipitation	0.56	0.09–2.40	0.485
Year	1.04	0.95–1.15	0.3988
Factors Multivariate	OR	95% CI for OR	*p* < 0.05
Cattle population	7.63	1.79–59.45	0.0172
Annual mean summer temperature (Jun-Aug)	2.86	1.16–9.71	0.0437

### Cattle population, mean summer temperature, and outbreak magnitude

**Fig 5:**
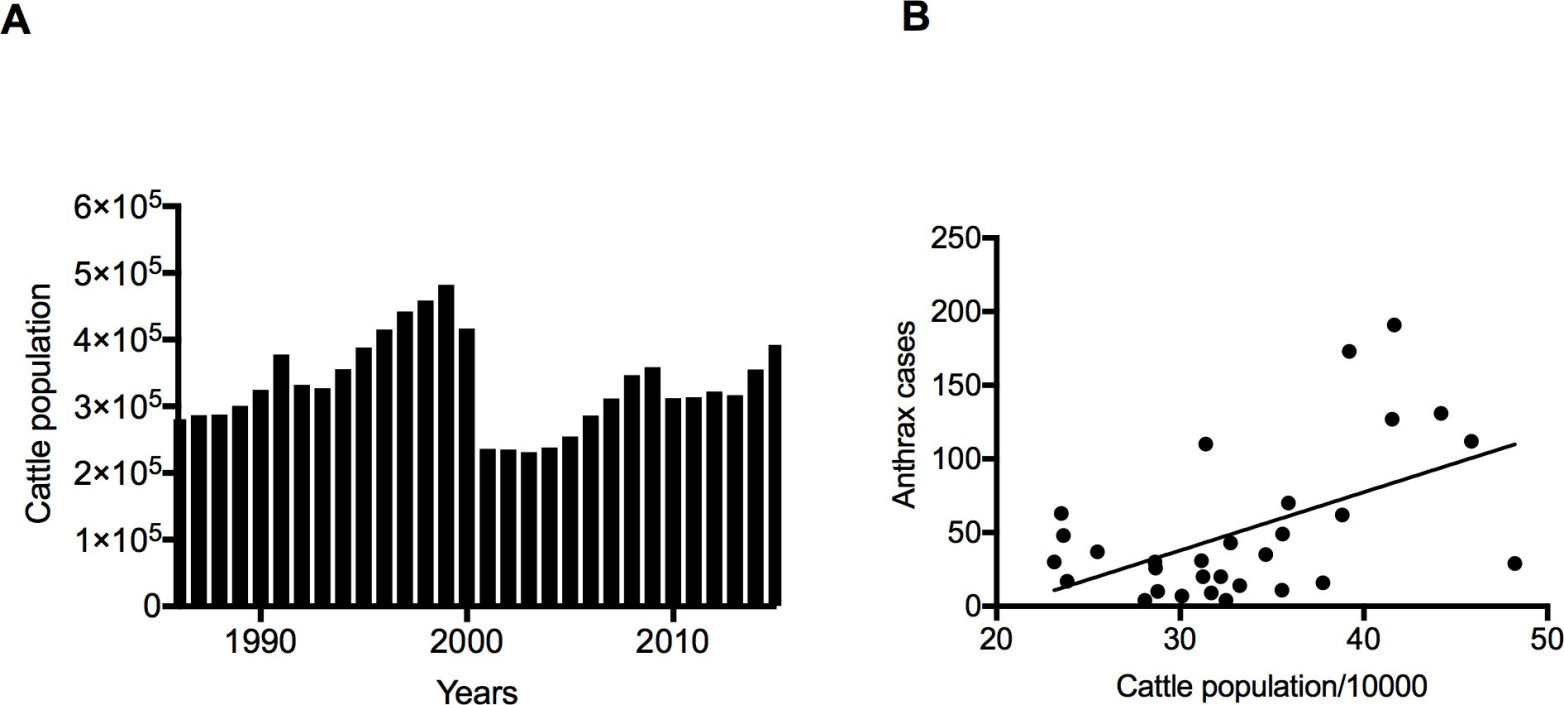
The magnitude of anthrax cases in relation to cattle population. (A) Cattle population by year. (B) Correlation between anthrax cases and cattle population between 1986 and 2015. r = 0.52, *p* = 0.003, 95% CI 0.2–0.743.

After identifying that cattle population and temperature are positively correlated with the anthrax case number, we investigated the extent to which the two factors affected the magnitude of an anthrax outbreak. We used a simple linear regression to model the dependence of anthrax cases on the cattle population over the entire 30-year period. We observed a steady increase in livestock population in the first half of the study period (1986–1999), followed by a decrease in 2000 before rising again in the second half (2001–2015) (Fig 5A). A drastic reduction in cattle number in 2000 was attributed to a disaster associated with severe climatic conditions, which is called zud in Mongolia. Mongolia was hit by three consecutive zuds between 1999 and 2002. Drought up to 60% of the national territory resulted in reduced pasture growth in summer and limited forage preparation by herders for the winter. Weakened by inadequate summer feeding and insufficient supplementary forage, several millions of animals died in extremely harsh winter with temperatures up to -50°C in some areas. Overall, the national livestock population decreased by about 12 million because of three-year sequent zud [37]. We found a significant relationship between anthrax cases and cattle population (r = 0.52, *p* = 0.003) (Fig 5B). The regression line’s slope was 3.95 ± 1.2, suggesting that for every 10,000 increase in cattle population size, the number of anthrax cases in cattle increased by an average of about 4.

**Fig 6:**
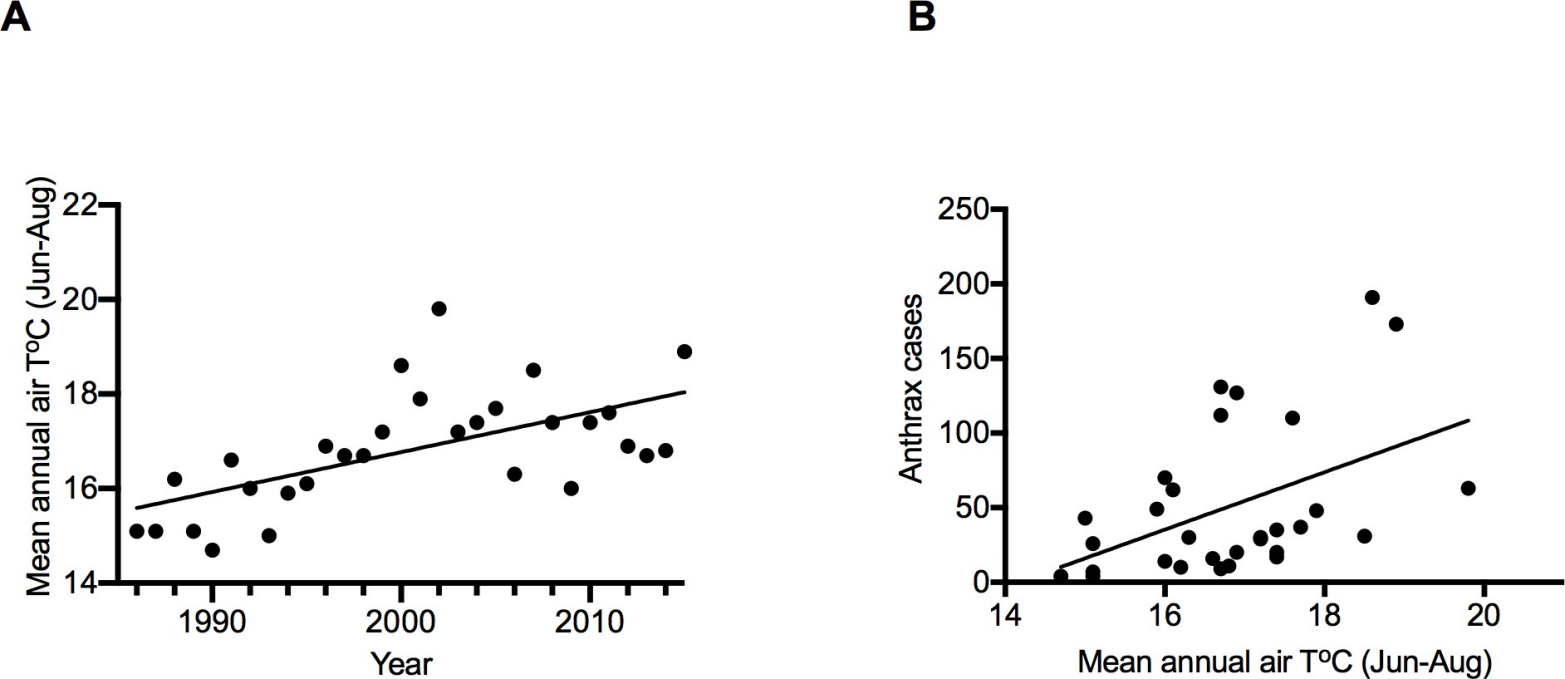
The magnitude of anthrax cases in relation to air temperature. (A) Linear regression of annual summer air temperature 1986–2015, slope = 0.08449 ± 0.02077, R^2^ = 0.4, *p* < 0.001, 95% CI 0.4195–0.127. (B) Correlation between anthrax cases and annual summer air temperature from 1986 to 2015 r = 0.46, *p* = 0.0099, 95% CI 0.1238–0.7058.

We observed a steady rise in annual mean summer temperature of 0.08449 ± 0.02077 (*p* < 0.001) (Fig 6A), which exhibited a positive correlation with the total anthrax cases in livestock over the entire study period (1986–2015) (r = 0.46, *p* = 0.009) (Fig 6B). The slope of the simple linear regression line was 19.24, suggesting that for every 1°C increase in air temperature, the number of livestock cases increased by 19. No positive correlation was observed between the cattle population and temperature changes (S3 Fig).

## Discussion

Here, we report spatial and temporal patterns of anthrax in livestock between 1986 and 2015 based on the carcass burial sites of animals that died of anthrax in Khuvsgul Province, showing the highest anthrax incidence rate in Mongolia. Our primary objective was to examine the spatio-temporal dynamics of the disease based on the carcass burial sites and identify where *B*. *anthracis* spores may persist in the present day. We first determined the spatial distribution of carcass sites in three historical periods (1986–1995, 1996–2005, 2006–2015) and found that the spatial distribution of carcass sites had not changed over the 30 years, indicating the recurrence of anthrax. Using KDE analysis on carcass sites, we identified two hotspots in low-lying areas around the south and north regions. There was a recently emerged hotspot identified in the northern part of the province in the last decade of the 30-year study period. Also, the highest proportion of anthrax cases was recorded in cattle, and cattle anthrax prevalence was high in several districts. Moreover, we showed the disease trend during and after the transition period of the country’s political change. Furthermore, we found a positive association between outbreak size and cattle population number and the mean annual air temperature of the summer months (June to August), highlighting the impact of these factors on anthrax occurrence in Khuvsgul region.

Results of spatial mean and SDE analyses revealed a localized source of exposure of anthrax. No significant changes were observed in the distribution of anthrax carcass sites for the entire 30 years. Unweighted spatial means and spatial overlapping of three SDEs corresponding to every decade overlaid in the same geographical area. It appears that animals are repeatedly exposed to spores in the same geographical areas. This suggests that historic carcass sites are a potential hazard that serves as a source of spore exposure, resulting in recurrent outbreaks. The localized source of exposure, defined as independent outbreaks recurring in the same geographical areas, was also identified in several other spatio-temporal studies on anthrax that characterized outbreaks following a point-source pattern with limited geographical spread [38–41]. Strong, consistent spatio-temporal patterns were observed in two anthrax outbreaks in hippopotamus (*Hippopotamus amphibious*) with 5-year intervals in the same location in the Queen Elizabeth Protected Area, Uganda [38]. In addition, consecutive episodes occurred in the same area with a high density of white-tailed deer (*Odocoileus virginianus*) carcasses in West Texas [39]. Furthermore, concurrent and spatially localized outbreaks were observed in the Serengeti ecosystem, Tanzania [17]. Known carcass sites could likely have been the source of anthrax infection over a certain distance range [9,42,43]. Therefore, proper disposal of carcasses is crucial to prevent further spore exposure from affected sites.

KDE analysis identified two anthrax hotspots based on the density of animal carcasses that died of anthrax (Fig 4). One hotspot has been detected around the southern region for the entire 30 years, whereas the second hotspot emerged in the northern region in the last decade of the study period. Interestingly, both hotspots were identified in low-lying areas around the Murun district in the south and the Darkhad Depression in the north. Regarding this particular geographical feature, Van Ness previously postulated that “anthrax incubator areas” retain high spore concentrations through the water cycle in a low-lying area or depression, resulting in spore doses that are lethal to a susceptible host and enough to trigger new outbreaks [14]. Supporting this postulate, carcass hotspots have been found only in low-lying areas that have wide rivers and lakes in our study (Figs 3 and 4). Notably, the Darkhad Depression encompasses many rivers, lakes, small potholes, and wetlands surrounded by mountains [44]. In agreement with our findings, Relebohile *et al*. [45], Bengis [46], and Dragon *et al*. [43] reported a high incidence of anthrax outbreaks in depressed low-lying areas. Although the effect of water area on the number of anthrax cases was not explored in this study, we suspect that it might increase the risk of many animals being infected from one spot. Thus, the disposal of affected animal carcasses near rivers and lakes should be avoided to restrict the spore dissemination and concentration through the hydrological cycle in a low-lying area. Besides, 30-year average human and livestock population densities were highest in Murun, the administrative center of Khuvsgul Province (S2 Fig). The pasture in this area could be overgrazed because of the high animal population density; thereby increasing the chance of animals ingesting or inhaling the spores. Also, anthropogenic pressure such as construction and agriculture may contribute to anthrax occurrence by exposing the spores to the ground surface, resulting in many carcass disposal sites. Therefore, human activities such as construction, mining, or agricultural development around carcass sites must be avoided and those areas must be secured by fencing to minimize future outbreaks.

Furthermore, we conducted univariate and multivariate logistic regression analyses to determine the potential association between the number of anthrax cases in livestock and possible risk factors. We found that the population size of susceptible animals and temperature increase are the factors that impact outbreak size. Other environmental and climatic drivers, including drought, rainfall, soil alkalinity, and density of insects or scavengers, have long been recognized as important factors influencing anthrax ecology [13]. In our study, however, precipitation including rainfall did not seem to impact the size of the outbreaks.

Cattle population number was determined as one of the risk factors for a large outbreak. From the univariate and multivariate logistic regression analyses, a significant positive correlation was found between the population number of cattle and annual anthrax cases from 1986 to 2015 (Table 3 and Fig 5B). A similar positive correlation between the number of anthrax cases and the hippopotamus population was detected in the Queen Elizabeth National Park, Uganda [38]. Hence, the susceptible animal number can be one of the determinants for outbreak size. Among the livestock species, cattle are most likely to contract anthrax in many cases [47,48]. From the studies conducted in Kazakhstan [49], China [50], and Ukraine [51], anthrax predominantly occurred in cattle compared with other animal species. In agreement with these reports, cattle anthrax occupied 76.5% of the total anthrax cases in Khuvsgul Province, and anthrax prevalence for 30 years was highest among cattle (Fig 1B and 1C), implying that cattle are the most susceptible to anthrax. Thus, in terms of resource-limited settings, cattle should be prioritized for vaccination. Moreover, cattle anthrax prevalence per 1 million population per 1000 km^2^ was high in the districts Murun, Chandmani-Undur, Khatgal, followed by Ikh-Uul, Tosontsengel, and Tsagaan-Uul (Table 2 and Fig 2). Therefore, more effort in vaccination and disease surveillance should be focused on these districts with a high burden of anthrax.

Also, increased temperature was detected as another risk factor. Recent studies emphasized the impact of global warming associated with permafrost melting on anthrax occurrence in northern latitudes of the globe [23]. After the reemergence of anthrax in reindeer over 70 years later in Yamal, Siberia, in 2016 [52] and Sweden [53], experts believed that infected animal carcasses previously buried in these regions were long preserved under the freezing effect of permafrost. However, permafrost melting resulting from global temperature increases the spore spillovers from the carcass into the ground surface, likely through moving sediments and soil cracking related to permafrost freeze–thaw activity [54,55]. This hypothesis is further supported by our findings, where an increasing trend of mean annual summer temperature showed a positive correlation with the number of anthrax cases in livestock in Khuvsgul (Fig 6A and 6B). The territory of Khuvsgul comprises a wide area of mountain permafrost, which is a continuation of the southern fringe of the Siberian permafrost zone, representing the highest permafrost prevalence in Mongolia [56]. From permafrost monitoring studies, the Khuvsgul region increased in mean annual permafrost temperature, coupled with intensive degradation of permafrost. These observations have been attributed to climatic factors such as global warming and anthropogenic elements like changing soil content, vegetation cover, and hydrologic cycle in the last several decades [36,57]. To this end, the long-term anthrax persistence and the highest incidence rate in the Khuvsgul Province could be explained by the ecosystem changes, the prevalence of permafrost, and its freeze–thaw dynamics. These observations suggest that permafrost may have a role in spore persistence in soil and *B*. *anthracis* infection cycle, making it a potentially useful spatial and temporal predictor of infection risk and anthrax outbreaks.

We could not obtain data on the animal anthrax vaccination trends, which is a major limitation of our study. But, a previous study showed that anthrax vaccination coverage decreased between 1990 and 2000 because of the country’s political revolution and economic transition phase [20]. This phase involved the privatizing of animal husbandry sectors and the suspension of veterinary services, resulting in a drastic drop in the anthrax vaccination coverage and a steady increase in anthrax cases. In agreement with the study, we observed a dramatic increase in yearly livestock anthrax cases in Khuvsgul Province from 1986 to 2000 (Table 1). Similar increasing anthrax trends were seen in other former Soviet countries, which was likely a result of socio-political instability [51,58]. Taken together, these findings highlight that Mongolia’s political change and economic transition affected anthrax occurrence, more likely through livestock vaccination. Since 2000, although neither a significant increasing nor decreasing trend was observed in the total livestock anthrax cases, cattle anthrax cases were significantly reduced, which had progressively increased during the transition period (S2 Table). We speculate that the downslope in cattle anthrax cases was likely associated with improving disease control measures, particularly cattle vaccination [20]. Further, extension of vaccination in livestock species that are excluded in routine vaccination programs is on demand for successful disease control in Mongolia, especially in the area with high-risk factors.

## Conclusions

We analyzed the spatio-temporal patterns of carcass burial grounds in Khuvsgul Province and found that livestock anthrax recurs in the same geographical areas. There is one stable hotspot of anthrax carcass sites around the south and an emerging new hotspot in the north region of the province. These hotspots exist in low-lying areas with abundant rivers, lakes, and ponds. Further, the burden of anthrax is higher in cattle than in other livestock species, and the cattle anthrax prevalence was high in the six districts. Finally, the size of outbreaks was influenced by the annual summer mean air temperature (June to August) of Khuvsgul Province, probably by affecting the permafrost freeze-thawing activity.

Our study suggests that historical carcass burial sites may serve as a persistent source of the infection. Thus, the primary action for carcass disposal management, we recommend fencing the old carcass sites to prevent possible animal spore exposure. Further, burying animal carcasses that died of anthrax should be banned and replaced by incineration, considering long-time preservation of *B*. *anthracis* spores in the frozen ground (permafrost) and its seasonal thawing effect on spore spillover to the soil surface. At least carcass disposal near rivers and lakes should be avoided to minimize spore dissemination and concentration through the hydrological cycle. As other control measures for anthrax, we suggest that strategical vaccination in susceptible animals, especially prioritizing cattle in the six districts mentioned above, due to the resource limitation. Another suggestion would be monitoring the permafrost condition in the endemic areas, which could be helpful to predict future outbreaks and preparing for epidemic responses such as strategic vaccination in susceptible animals. Future work should involve detailed field surveys on spore viability around the carcass burial sites and molecular epidemiology of the *B*. *anthracis* strains on isolates.

## Supporting information

S1 Fig Anthrax in livestock species. (TIF)

S2 Fig Average human and livestock population densities by districts of Khuvsgul Province (1986–2015). Murun district is the administrative center of the province and is estimated with the highest human and livestock population densities. The maps are reprinted from [28] under a CC BY license, with permission from DIVA-GIS and Dr. Robert Hijmans; see S1 File.
(TIF)

S3 Fig Interaction between temperature and cattle population. There was no correlation observed between the two risk factors with r = −0.0389, *p* = 0.83, 95% CI −0.3936–0.3259.
(TIF)

S1 Table Result of multi-distance spatial cluster analyses. The maximum expected K distances with statistically significant values are highlighted with a gray background in the table. The spatial values corresponding to the time phases were then used for kernel density estimation analysis on anthrax carcass sites.
(XLSX)

S2 Table Annual cattle anthrax incidences in Khuvsgul Province during (1986–2000) and after (2001–2015) the economic transition of Mongolia. (XLSX)

S1 File Permission to publish map shapefiles. (PDF)
