# Supplementary figures and images for: Risk factors and spatio-temporal patterns of livestock anthrax in Khuvsgul Province, Mongolia
Source: PLoS One. 2021 Nov 19;16(11):e0260299. doi: 10.1371/journal.pone.0260299 (PMC8604359; doi:10.1371/journal.pone.0260299)

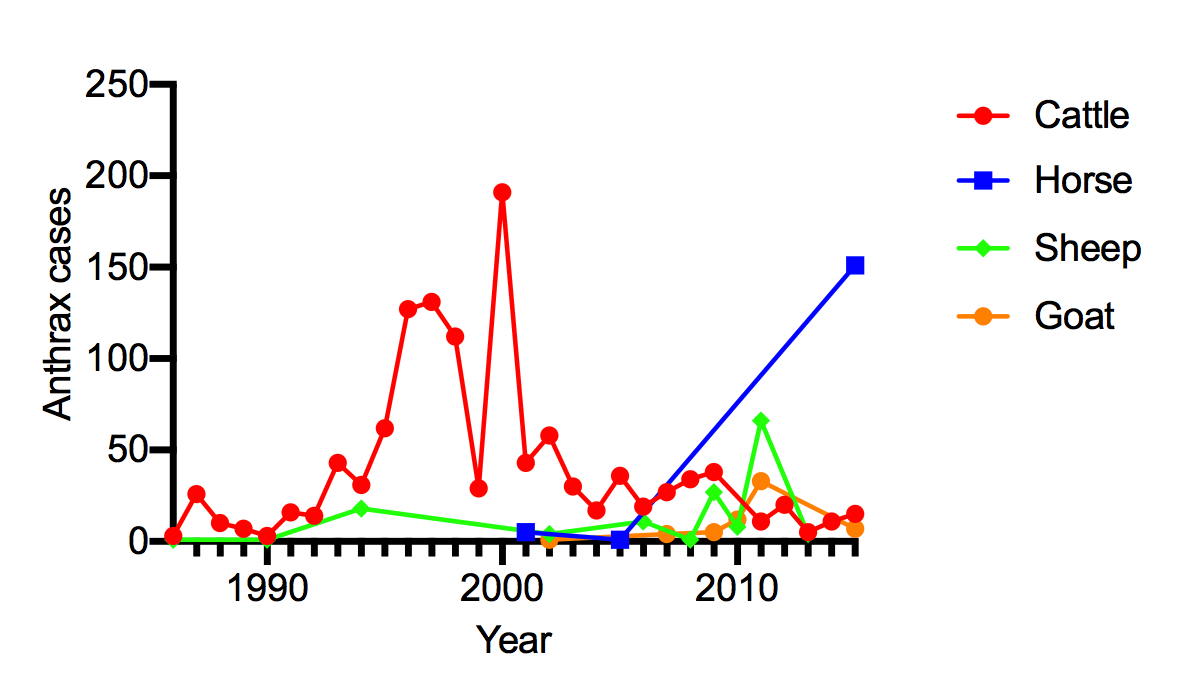

Supplement: S1 Fig — (TIF) [file pone.0260299.s001.tif]

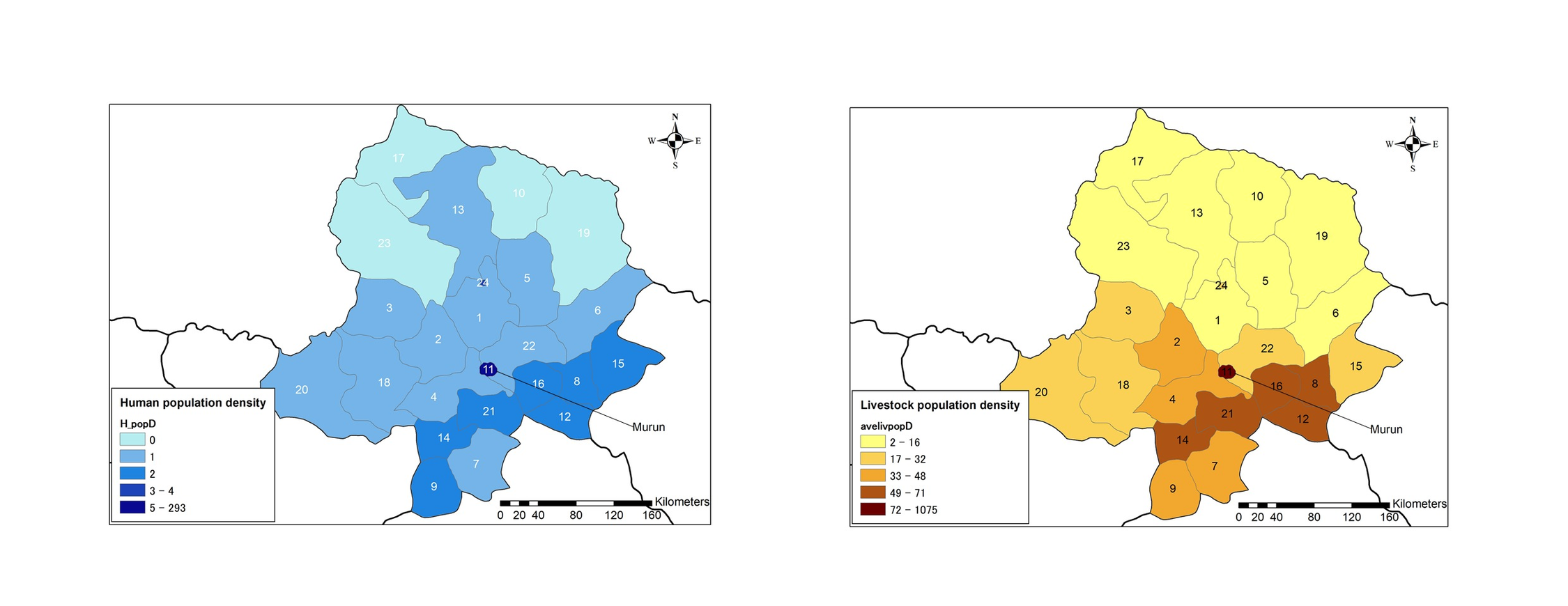

Supplement: S2 Fig — Murun district is the administrative center of the province and is estimated with the highest human and livestock population densities. The maps are reprinted from [28] under a CC BY license, with permission from DIVA-GIS and Dr. Robert Hijmans; see S1 File. (TIF) [file pone.0260299.s002.tif]

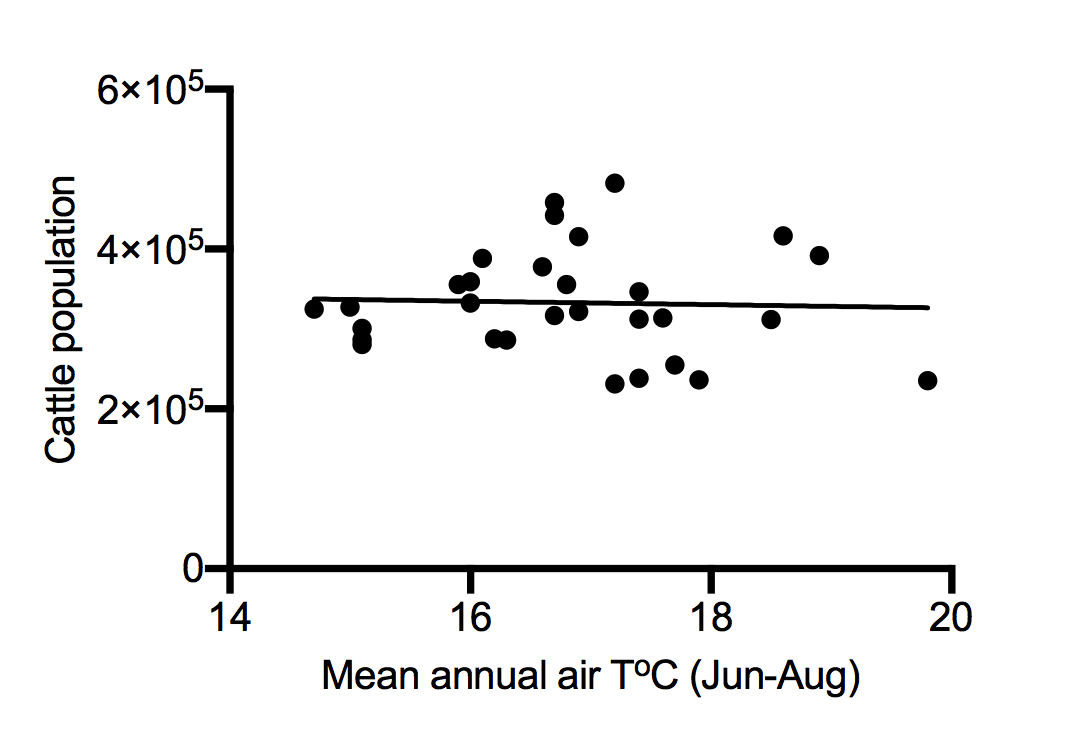

Supplement: S3 Fig — There was no correlation observed between the two risk factors with r = −0.0389, p = 0.83, 95% CI −0.3936–0.3259. (TIF) [file pone.0260299.s003.tif]
