# Supplementary material for: Risk factors and spatio-temporal patterns of livestock anthrax in Khuvsgul Province, Mongolia
Source: PLoS One. 2021 Nov 19;16(11):e0260299. doi: 10.1371/journal.pone.0260299 (PMC8604359; doi:10.1371/journal.pone.0260299)
Supplement: S1 File — (PDF) [file pone.0260299.s006.pdf]

Request for Permission to Publish Content under CC-BY License

Dear Rights Holder or Representative,

I have submitted a paper for publication in a PLOS journal, and wish to include the content listed below in the paper I'm hereby requesting your permission to include the content in my paper. Please note that all PLOS journals are published under a Creative Commons Attributions License (CC BY), which allows for unrestricted use and distribution, even commercial, as long as attribution is given to the creator or rights holder of the content. See https://creativecommons.org/licenses/by/4.0/.

To grant me permission to use the content in my PLOS paper, please fill in the information below and then scan the completed form and send it to me at my email address.

Thank you.

My name:

Tuvshinzaya Zorigt (supervisor Hideaki Higashi)

My email address:

tuvshinzaya@czc.hokudai.ac.jp (hidea-hi@czc.hokudai.ac.jp)

Description of the content which I'm seeking permission to use:

Shapefiles for Mongolia; Administrative areas, Elevation, and Inland Water

Link to the content:

https://diva-gis.org/gdata

***

On behalf of myself or the right holder, I hereby grant the permission sought herein.

Signature of Party Granting Permission:

Date:

August 16, 2021

Printed Name and Title

Robert Hijmans, owner
